# Optimization of UV-C Processing of Donkey Milk: An Alternative to Pasteurization?

**DOI:** 10.3390/ani11010042

**Published:** 2020-12-28

**Authors:** Photis Papademas, Panagiotis Mousikos, Maria Aspri

**Affiliations:** Dairy Science and Technology, Department of Agricultural Sciences Biotechnology and Food Science, Cyprus University of Technology, 3036 Limassol, Cyprus; panagiotis.mousikos@cut.ac.cy (P.M.); maria.aspri@cut.ac.cy (M.A.)

**Keywords:** donkey milk, UV-C technology, non-thermal methods, microbial inactivation, milk pathogens

## Abstract

**Simple Summary:**

Donkey milk has received much interest lately due to its chemical composition, which is very close to human milk as well as to its unique functional properties (antibacterial, antioxidant, immunomodulatory, and antitumor activities). Furthermore, donkey milk is considered a valid alternative milk for infants and adults suffering from cow milk protein allergy. However, it is recommended by pediatricians and clinicians that raw donkey milk must be thermally processed to render it safe for sensitive population (i.e., infants and immunocompromised). On the other hand, thermal processing is known to reduce the bioactivity of milk. Consequently, the objective of this study is to determine the feasibility of the UV-C system to inactivate or reduce foodborne pathogens in raw donkey milk in order to produce a safe, non-thermally processed donkey milk that can be consumed by special population groups (infants, elderly, immunocompromised). Results obtained from this study indicate that UV-C has the potential to be used as a non-thermal treatment to reduce food borne pathogens present in raw donkey milk.

**Abstract:**

The effect of UV-C light technology on the inactivation of six foodborne pathogens inoculated in raw donkey milk was evaluated. Fresh raw donkey milk was artificially inoculated with the following foodborne pathogens—*L. inoccua* (NCTC 11288), *S. aureus* (NCTC 6571), *B. cereus* (NCTC 7464), *Cronobacter sakazakii* (NCTC 11467), *E. coli* (NCTC 9001), *Salmonella enteritidis* (NCTC 6676)—and then treated with UV-C doses of up to 1300 J/L. *L. innocua* was the most UV-C-resistant of the bacteria tested, requiring 1100 J/L for complete inactivation, while the rest of the bacteria tested was destructed in the range of 200–600 J/L. Results obtained from this study indicate that UV-C light technology has the potential to be used as a non-thermal processing method for the reduction of spoilage bacteria and foodborne pathogens that can be present in raw donkey milk.

## 1. Introduction

In recent years, there has been a growing interest for donkey milk production and commercialization due to its similar composition with human milk, making it ideal for consumption by sensitive population groups such as infants with cow milk protein allergy (CMPA), the immunocompromised, and the elderly [1]. According to the literature, raw donkey milk has a low total bacterial count; nevertheless, the presence of foodborne pathogens such *as E. coli 0157*, *S. aureus*, *Campylobacter* spp., and *Cronobacter sakazaki* was also detected [2,3,4,5]. Therefore, raw donkey milk is subjected to thermal pasteurization in order to ensure its microbiological safety and to comply with the European Community Regulation 853/2004. However, the method has many drawbacks such as high energy cost, and it may influence protein denaturation, deterioration of the technological properties of milk (e.g., prolong enzymatic milk protein coagulation) and loss of nutrients, which is associated with flavor degradation. Thus, there is an interest for alternative methods that would ensure the microbiological safety of the milk while preserving its high biological value. UV-C light technology can be used as an alternative non-thermal method.

Ultraviolet light (UV-C) treatment is one of the most promising non-thermal technologies to destruct microorganisms in milk without involving heat [6,7,8,9,10,11,12,13,14,15,16]. UV technology has the advantage over thermal pasteurization of its ability to minimize the loss of flavors and nutrients, and it is more energy efficient. A limitation of UV-C light processing of milk is its ability to penetrate into opaque liquids resulting in no or very low microbial inactivation [17]. Therefore, a strategy to increase the penetration is the use of turbulent flow reactors so that liquid foods are exposed to UV light uniformly.

UV radiation covers part of the electromagnetic spectrum in the range of 100–400 nm, which is categorized into three ranges based on their photochemical propertied and biological effects: UV-A (315–400 nm), UV-B (280–315 nm), and UV-C (200–280 nm) [6,18]. Microbial inactivation from UV light is associated with photochemical changes that take place in proteins and nucleic acids within the cell membrane when UV light is absorbed by the food during UV-C treatment. Photons interact with thymine and cystine nucleoside bases, causing the formation of cross-linked photoproducts, especially cyclobutyl pyrimidine dimers (CPD), which disrupt the DNA transcription, translation, and replication processes that lead to the loss of microbial cell functions and ultimately to cell death of the microorganism [18,19]. The UV-C light, in particular the wavelength range 250–260 nm, has optimal properties for the inactivation of bacteria, yeast, bacterial spores, molds, and viruses and is most widely used in the food processing industry [10].

The objective of this study was to determine the feasibility of a continuous UV-C system to inactivate or reduce foodborne pathogens that were artificially inoculated in raw donkey milk compared to the conventional pasteurization process.

## 2. Materials and Methods

### 2.1. Collection of Milk Samples

Milk samples were collected from the “Golden Donkeys Farm”, located in Larnaca district, Cyprus. All donkeys were fed the same diet consisting of hay, barley, corn, and a concentrate of minerals, vitamins, and salt following the European Directive 98/58/EC. Donkeys were healthy, and no antibiotics were administrated prior to sampling. The process of milking was carried out in the stable, and donkeys were milked manually from the same milker. During milking, the udder was cleaned using sterile wet wipes and the nipples using 70% ethanol and dried with sterile gauze. Sampling for microbiological and physicochemical analysis was conducted weekly (33 weeks) from October of 2018 until May 2019 from the daily milk batch (20 L from 20–25 milking donkeys). The donkey milk samples were collected in sterilized containers (250 mL), placed in cool-boxes, and transported to the laboratory (4 °C) and processed during the same working day for physicochemical and microbiological analysis. These 33 samples were used to assess the general microbiological quality of raw donkey milk.

For the UV-C and pasteurization experiments, three batches of 7 L donkey milk samples were collected as described above. The initial background flora of the raw milk was assessed each time before subsequent inoculation and processing (UV-C or pasteurization) by serial dilutions in saline solution. A series of dilutions were pour plated in duplicate on Plate Count Agar, and the plates were incubated at 30 °C for 72 h.

### 2.2. Bacterial Strains

All strains were obtained from National *Collection* of Type Cultures (NCTC). *L. inoccua* (NCTC 11288), *S. aureus* (NCTC 6571), *B. cereus* (NCTC 7464), *Cronobacter sakazakii* (NCTC 11467), *E. coli* (NCTC 9001), and *Salmonella enteritidis* (NCTC 6676) were reconstituted and growth at 37 °C in brain heart infusion (BHI) broth. All cultures were stored in glycerol (20% vol/vol) at −80 °C until use. The selection of the above pathogens was based on the fact that these are the main foodborne pathogens associated with milk and dairy products contamination. Moreover, *B. cereus* is a Gram-positive spore-forming bacterium that is capable of surviving pasteurization temperatures, while *Cronobacter sakazakii* has been mainly isolated from dried infant formula, and also, both strains has been previously isolated from raw donkey milk [3]. Therefore, the selection of the above strains was based on the future applications of this study, which is the production of a UV-C freeze-dried donkey milk powder and also taking into account the main target group of consumers (i.e., infants, immunocompromised, and the elderly).

### 2.3. Growth of Bacterial Strains and Milk Inoculation

To determine the log reduction capability of UV-C and pasteurization treatments, raw donkey milk samples were artificially inoculated with a cocktail of the above bacterial strains at a final concentration of approximately 5 log CFU/mL. Fresh overnight cultures of all the strains were added directly to donkey milk (7 L) and mixed for 5 min. After 5 min, 500 mL of the artificially inoculated raw donkey milk was placed into a beaker to be used for the pasteurization experiment. Background populations of the pathogens mentioned above were not detected in donkey milk samples prior to inoculation.

### 2.4. UV-C Processing of Artificially Contaminated Raw Donkey Milk

**Figure 1:**
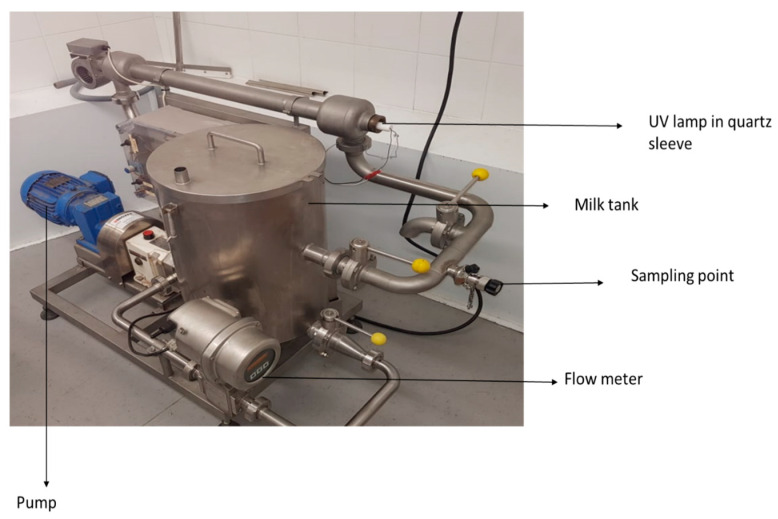
SurePure SP1 UV system.

Ultraviolet treatment of artificially inoculated donkey milk (7 L) was performed in a pilot-scale, low-power UV unit designated SP-1 (SurePure) (Figure 1). The unit contained a UV bulb enclosed in an optically pure quartz sleeve that separates milk from the light source. The ‘‘SurePure Turbulator™’’ UV-C device creates turbulent flows and is designed for continuous flow inactivation of turbid fluids such as milk. The SP-1 unit was operated at a flow rate of 4000 L/h (1.11 L/s). The following UV doses were used: 0, 91.8, 275.4, 459, 642.6, 826.2, 1000.8, 1100, 1200, and 1300 J/L. The ultraviolet light intensity at the surface of the quartz sleeve was 17.7 mW/cm^2^ according to the UV lamp manufacturer. According to manufacturer specifications, the turbulator had a unit area of A = 7.85 × 104 m^2^ (d1 = 24.5 mm, d2 = 40 mm, where d1 and d2 are the inner and outer diameters, respectively of the turbulator), a volume of 0.675 L, and residence time of 0.608 s in a single unit. Therefore, at a flow rate of 4000 L/h, 25.2 s are required for 7 L of donkey milk to pass through the reactor once; thus, one turn of the product through the system is equivalent to a UV-C dose of 22.95 J/L. The UV dosage per L of donkey milk treated for one reactor with continuous flow was calculated as follows: Dosage = Total UV-C output per unit (W)/Flow rate (L/s) = 25.50 W/1.11 L/s = (25.50 J/s)/(1.11 L/s) = 22.95 J/L. Moreover, the UV dosage per surface area for one reactor was calculated as follows: UV dosage per area = UV intensity (I) on the surface of the sleeve × residence time (*t*); 17.7 mW/cm^2^ × 0.608 s = 10.75 mJ/cm^2^. Samples (50 mL) were taken from an aseptic sampling port at specific time points corresponding to each UV dose in order to assess the microbial reduction. The SP-1 UV system was cleaned and sanitized after each use with a clean-in-place process. The process was repeated three times.

### 2.5. Donkey Milk Pasteurization (Control)

A subsample of 500 mL of the artificially inoculated raw donkey milk to be used for UV-C treatment was placed into a glass beaker with a magnetic stirrer and heated on a hotplate. A thermometer was immersed into the milk to monitor the temperature during the pasteurization process. Heat treatment has been carried out at 62.5 °C for 30 min, which is the gold standard treatment of a (milder) heat processing of donor human milk [20]. After pasteurization, the donkey milk was quickly cooled down in an ice bath until the temperature reached 4 °C [21].

### 2.6. Microbiological Analysis

UV-C treated, pasteurized, and untreated (milk before inoculation of target organisms) donkey milk samples were 10-fold diluted and spread or pour plated in duplicate on the appropriate media and incubated at specific conditions for each bacterial strain, as shown in Table 1.

For higher UV dosage, undiluted samples were spread plated (1 mL across 3 plates) on appropriate agar for more precise microbial counts. The colonies were counted to determine the colony-forming units per milliliter. Following incubation, CFUs from triplicate plates were counted. In order to determine the reduction in bacterial number during UV-C treatment, CFU/mL values were averaged and converted into logarithmic units because the dairy microbiological date does not usually follow a normal distribution.

### 2.7. Lethality Calculations

Microbial counts (cfu/mL) were converted to log10 and then plotted as a function of UV-C dose.

Mathematical models used to explain the inactivation kinetics of microorganisms were analyzed by a freeware add-in GInaFiT (version 1.6) using Microsoft Excel 2010 [22]. Results were the mean of three different batches. For each pathogen, three inactivation models were tested (Table 2).

The goodness of the fit of each model was evaluated using the values of higher regression coefficient (R^2^), and the lower root mean square error (RMSE). GInaFiT also provides the parameter 4D, which is defined as the treatment dose necessary to inactivate 99.99% of the microbial population.

## 3. Results

**Figure 2:**
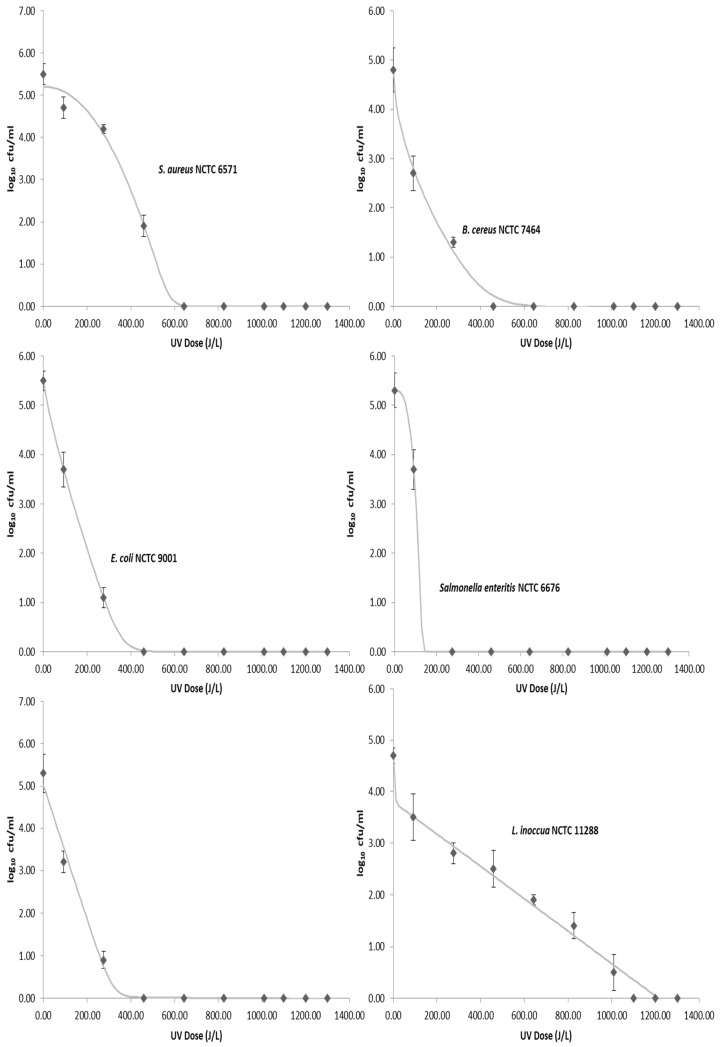
Survival curves for each pathogen. Points are the mean value and error bars represent standard deviation. The lines represent the inactivation model generated using the GInaFit software.

The average initial concentration of the foodborne pathogens inoculated into the donkey milk ranged from 4.7 to 5.5 log CFU/mL. None of the foodborne pathogens were detected in any of the donkey milk samples prior to inoculation, while the average total viable count (TVC) was 2.7 log CFU/mL. Results showed that sensitivity of foodborne pathogens to UV-C light can vary significantly among genera (Figure 2). Microbial counts of bacteria decreased as the cumulative dose of UV-C light increases. Among the tested organisms, *L. inoccua* proved to be the most resistant to UV-C treatment. *S. enterica*, *B. cereus*, *E. coli*, and *Cronobacter sakazakii* reacted similarly to exposure to UV-C irradiation and required similar dosages to obtain a 5-log10 reduction, while *S. aureus* required 459 J/L for complete inactivation. At a UV-C irradiation dose of 1100 J/L, no bacterial growth was observed for all the tested strains. Moreover, results of the physicochemical and microbiological quality of raw donkey milk as sampled for a period of 33 weeks are presented in Table 3. Heat treatment of artificially inoculated donkey milk at 62.5 °C for 30 min was able to reduce the population of foodborne pathogens to not detectable levels.

The survival data of all foodborne pathogens used in the study after each UV-C dose were adjusted to different linear and nonlinear models of survival curves (linear, Weibull, biphasic) using the GInaFiT software (Microsoft^®^ Excel). As previously mentioned, the root mean square error (RMSE) and the R^2^ value were used to determine the fitting accuracy. Therefore, using the RMSE and R^2^ values calculated for each inactivation kinetic model as an indicator of goodness of fit, the biphasic model was the best fit for the UV-C inactivation data for *L. inoccua* and *Cronobacter sakazakii*, while the double Weibull model was the best fit for *S. aureus, B. cereus, E. coli,* and *Salmonella enteritis* (Table 4).

Moreover, in this study, the UV-C dose necessary to reduce 4 log CFU/mL (4D) of the initial bacterial populations was calculated using the best fit inactivation model for each foodborne pathogen (Table 5).

## 4. Discussion

The UV-C system (turbulent flow) used in this study was able to reduce the bacterial population to not detectable levels by ensuring that the total volume of donkey milk is exposed to the surface of the light source. A mixture of different bacterial strains was inoculated into raw donkey milk in order to test the validity of UV-C, which is more representative of the multi-strain nature of microbial contamination in milk [27]. The SurePure UV-C system used in this study was also used in similar studies, and it was proven that is able to reduce pathogens and spoilage flora present in milk, fruit juices, and wine [10,12,15,28,29]. The fact that between studies, different parameters are present such as (a) type of UV-C equipment employed, (b) bacterial species, (c) processing conditions, and (d) physical properties of the matrices make it difficult to compare results.

A study carried out by [12] showed that the exponential reduction had a high correlation with the applied UV-C dosage. Moreover, the decimal reduction dosage for *E. coli, B. cereus,* and *S. aureus* was between 594 and 640 J/L, which is a little bit higher than the UV-C dosage found in this study. However, this difference may be due to the different UV-C equipment and bacterial cultures used in both studies. Moreover, the amount of total solids can also affect the UV-C efficiency. The human milk used in [12] study has a total solid concentration of 10.5–14.5 g/100 mL, which is higher than that of donkey milk (9.23 ± 0.29 g/100 mL).

Regarding *L. monocytogenes*, several authors [6,7,9,10,28,30] have shown that it is more UV resistant when compared to other foodborne pathogens inoculated in milk, which is accordance with the results of this study. This fact can be attributed to the thicker peptidoglycan cell wall of Gram + ve bacteria in comparison to Gram -ve bacteria, which can hinder the penetration of UV photons within bacterial cells as well as to the ability of *L. monocytogenes* to cope with DNA damages by having a more efficient DNA repair mechanism in comparison to other foodborne pathogens such as *E. coli* [31,32,33]. For instance, [7,10,30] showed that for a 5-log reduction of *L. monocytogenes* in Ultra High Temperature (UHT) full-fat milk, a UV-C dose of 2000 J/L is required, while [7,10,30] reported that a UV dose of 15.8 mJ/cm^2^ led to more than 5 log reduction in *L. monocytogenes* in goat milk. The UV-C dose required for the reduction of *L. monocytogenes* by 5 log was slightly higher that the UV-C dose found in this study. This could be due to the differences in composition of donkey milk, goat, and bovine milk in terms of fat and total solids.

Mathematical models that accurately explain the inactivation kinetics of foodborne pathogens in donkey milk may assist in the successful implementation of UV-C technology at the industrial level, as well as on the understanding the effect of a number of factors that should be taken into account for process optimization [34,35]. The results of the inactivation studies are in agreement with previous studies that showed that the inactivation of microorganisms do not follow first-order kinetics, especially for non-thermal processes [10,34,36]. The mixed Weibull model assumes the existence of two subpopulations—a more sensitive and a more resistant—while biphasic inactivation curves result from a microorganism with heterogeneous resistance to the inactivating agent [10]. The initial rapid decrease in population demonstrates the death of the less resistant strain, while the second part of the biphasic curve represents the death of resistant strains [27].

In addition, the calculation of D value can be of great importance for food industries planning to use UV-C technology in order to evaluate the necessary UV-C dose for the inactivation of microorganisms. However, in most cases using UV-C, inactivation kinetics of microorganisms do not follow first order, hence making it difficult to estimate the D-value required to reduce 90% of the microbial population.

## 5. Conclusions

In summary, this is a primary study demonstrating that UV-C treatment could offer an effective alternative to pasteurization in order to reduce the bacterial population of donkey milk to acceptable limits. The results showed that UV-C was effective in reducing the microbial population in the artificially contaminated donkey milk. Nevertheless, further studies are underway to investigate the effect of UV-C treatment on the biologically active components such as immunoglobulins, lactoferrin, and lysozyme as well as on vitamins (i.e., vitamins C and D). Additionally, the effect on the digestibility and bioavailability of nutrients that processing has on the in vitro digesta of milk samples will be evaluated. Finally, peripheral blood mononuclear cell lines will be utilized in order to assess any effect that donkey milk digesta (and the type of processing) has on stimulating cells (i.e., cytokines) responsible for any immune-regulating effect.

## Figures and Tables

**Table 1:** Bacterial growth conditions.

Microbiological Parameter	Microbiological Media	Incubation Conditions	Reference
*S. aureus* NCTC6571	Baird Parker	37 °C/48 h	ISO 6888-1:1999
*L. inoccua* NCTC 11288	ALOA	37 °C/48 h	ISO 11290-2:2017
*B. cereus* NCTC 7464	MYP	30 °C/48 h	ISO 7932:2004
*E. coli* NCTC 9001	TBX	44 °C/24 h	ISO 16649-2:2001
*Salmonella enteritis* NCTC 6676	Chromogenic Salmonella	37 °C/48 h	ISO 6579-1:2017
*Cronobacter sakazakii* NCTC 11467	Chromogenic	44 °C/24 h	ISO 22964:2017

**Table 2:** Microbial inactivation models.

Inactivation Model	Equation	Reference
Log Linear Model	$\log_{10}\left(N\right)=\log_{10}\left(N_{0}\right)-\left(\frac{kmax*t}{Ln\left(10\right)}\right)$	[23]
Weibull Model	$\log_{10}\left(N\right)=\log_{10}\left(N_{0}\right)-{\left(\frac{t}{\delta }\right)}^{p}$	[24]
Double Weibull Model	$\log_{10}N=\log_{10}\frac{10^{N_{0}}}{1+10^{\alpha }}\left[10^{{-}^{{\frac{t}{\delta_{1}}}^{p+a}}}+10^{{-}^{{\frac{t}{\delta_{2}}}^{p}}}\right]$	[25]
Biphasic Model	$\log_{10}N=\log_{10}N_{0}+\log_{10}\left(f*e^{-k_{max1}t}+\left(1-f\right)*e^{-k_{max2}t}\right)$	[26]

**Table 3:** Microbiological and chemical quality of raw donkey milk (n = 33).

Microbiological Parameters (log cfu/mL)	Min	Max	Mean	SD
TVC	2.90	5.10	3.80	0.02
Enterobacteriaceae	<1.00	3.40	1.90	0.04
Staphylococcus	<1.00	4.70	3.10	0.06
*E. coli*	<1.00	<1.00	<1.00	<1.00
*Bacillus cereus*	<1.00	<1.00	<1.00	<1.00
*Listeria monocytogenes*	ND	ND	ND	ND
**Chemical Parameters (g/100 mL)**				
Fat	0.30	1.40	0.84	0.07
Protein	1.30	1.96	1.62	0.05
Total Solids	7.29	10.59	9.23	0.69

**Table 4:** Root mean square error (RMSE) and R2 measures for each pathogen using different inactivation models.

Inactivation Model	Log Linear		Weibull		Double Weibull		Biphasic	
Bacteria	RMSE	R^2^	RMSE	R^2^	RMSE	R^2^	RMSE	R^2^
*S. aureus*	1.00	0.83	0.94	0.87	0.21	0.99	0.42	0.98
*L. inoccua*	0.32	0.97	0.27	0.98	0.29	0.98	0.23	0.99
*B. cereus*	1.25	0.50	0.83	0.80	0.12	0.99	0.30	0.98
*E. coli*	1.30	0.61	0.85	0.85	0.0096	1.00	0.11	0.99
*Salmonella enteritis*	1.43	0.52	1.27	0.67	0.0008	1.00	0.12	0.99
*Cronobacter sakazakii*	1.25	0.59	0.96	0.78	0.64	0.92	0.20	0.99

**Table 5:** 4D values for each pathogen using the best fit inactivation model.

Pathogen	Inactivation Model	4D Value (J/L)
*S. aureus*	Double Weibull	507
*L. inoccua*	Biphasic	1001
*B. cereus*	Double Weibull	338
*E. coli*	Double Weibull	247
*Salmonella enteritis*	Double Weibull	130
*Cronobacter sakazakii*	Biphasic	260
